# Identification and validation of obesity-related gene *LEP* methylation as a prognostic indicator in patients with acute myeloid leukemia

**DOI:** 10.1186/s13148-021-01013-9

**Published:** 2021-01-23

**Authors:** Ting-juan Zhang, Zi-jun Xu, Yu Gu, Ji-chun Ma, Xiang-mei Wen, Wei Zhang, Zhao-qun Deng, Jun Qian, Jiang Lin, Jing-dong Zhou

**Affiliations:** 1 Department of Hematology, Affiliated People’s Hospital of Jiangsu University, 8 Dianli Rd., Zhenjiang, 212002 People’s Republic of China; 2 Zhenjiang Clinical Research Center of Hematology, Zhenjiang, Jiangsu People’s Republic of China; 3 The Key Lab of Precision Diagnosis and Treatment in Hematologic Malignancies of Zhenjiang City, Zhenjiang, Jiangsu People’s Republic of China; 4 Laboratory Center, Affiliated People’s Hospital of Jiangsu University, 8 Dianli Rd., Zhenjiang, 212002 People’s Republic of China

**Keywords:** Obesity, *LEP*, Methylation, Prognosis, AML

## Abstract

**Background:**

Obesity confers enhanced risk for multiple diseases including cancer. The DNA methylation alterations in obesity-related genes have been implicated in several human solid tumors. However, the underlying role and clinical implication of DNA methylation of obesity-related genes in acute myeloid leukemia (AML) has yet to be elucidated.

**Results:**

In the discovery stage, we identified that DNA methylation-associated *LEP* expression was correlated with prognosis among obesity-related genes from the databases of The Cancer Genome Atlas. In the validation stage, we verified that *LEP* hypermethylation was a frequent event in AML by both targeted bisulfite sequencing and real-time quantitative methylation-specific PCR. Moreover, *LEP* hypermethylation, correlated with reduced *LEP* expression, was found to be associated with higher bone marrow blasts, lower platelets, and lower complete remission (CR) rate in AML. Importantly, survival analysis showed that *LEP* hypermethylation was significantly associated with shorter overall survival (OS) in AML. Moreover, multivariate analysis disclosed that *LEP* hypermethylation was an independent risk factor affecting CR and OS among non-M3 AML. By clinical and bioinformatics analysis, *LEP* may be also regulated by *miR-517a/b* expression in AML.

**Conclusions:**

Our findings indicated that the obesity-related gene *LEP* methylation is associated with *LEP* inactivation, and acts as an independent prognostic predictor in AML.

## Background

Acute myeloid leukemia (AML) is an aggressive hematological malignancy characterized by clonal proliferation of the hematopoietic progenitor cells [1]. Clinical outcome of AML is heterogeneous due to the cytogenetically and molecularly diverse [1]. Despite the improved treatment regimens, more than 50% of AML patients experience short-term recurrence [1]. Early identification of patients with poor prognosis, and then given intervene accordingly can help to improve AML survival. Currently, the 2017 European LeukemiaNet (ELN) risk stratification by genetics is widely accepted, but there is a practical limitation to the definition of genetic risk, especially in patients falling in the intermediate group [2]. Therefore, additional prognostic factors are needed.

Obesity has been verified as an independent health risk and is significantly correlated with the development of metabolic disorders, including hyperlipidemia, type 2 diabetes mellitus, hypertension, stroke, and cardiovascular disease. Furthermore, strong evidences have proved the links between body mass index (BMI) and various cancers including the most forms of tumor-based cancer and hematological malignancies [3–6]. A number of studies have showed the increased risk of cancer including leukemia incidence in obese patients [7, 8], and excess fat mass is associated with both enhanced incidence and lower survival for pediatric leukemia [9]. Moreover, obesity independently conferred poor prognosis in AML [10]. Yan et al. [11] revealed that Fatty acid-binding protein 4 mechanistically linked obesity with aggressive AML by enhancing aberrant DNA methylation. To date, various genes such as *LEP*, *LEPR*, *NPY*, *ADIPOQ*, *FTO*, *MC4R*, *PCSK1*, and *POMC* are implicated and have a direct role in obesity [12].

To the best of our knowledge, epigenetic alterations have been suggested as a molecular mechanism mediating gene expression, and also described as a potential early cancer-related biomarker with strategies for diagnostic, prognosis or cancer screening procedures being developed [13, 14]. To date, epigenetic mechanisms including DNA methylation and aberrant microRNAs (miRNAs) expression involving in obesity-related genes have been reported especially in human cancers with prognostic significance [12, 15]. However, the underlying role and clinical implication of DNA methylation of obesity-related genes in AML has yet to be elucidated.

## Materials and methods

### Patients and samples

The first cohort of 200 AML patients from The Cancer Genome Atlas (TCGA) databases included in this study was used in the discovery stage for the identification of prognostic methylation-related genes [16]. Among the cohort, there are 173 cases with expression data whereas 194 patients with methylation data [17, 18]. In addition, DiseaseMeth version 2.0 (http://bio-bigdata.hrbmu.edu.cn/diseasemeth/analyze.html) was applied to compare the methylation difference between these AML patients with controls.

A second cohort of 25 healthy donors and 111 de novo AML patients treated at the Affiliated People’s Hospital of Jiangsu University were also enrolled, and used in the validation stage for targeted bisulfite sequencing. In addition, expanded samples of the sequencing cohort, including 172 AML patients (161 de novo AML and 11 MDS-derived AML) and 46 healthy donors, were used in the validation stage for real-time quantitative methylation-specific PCR (qMSP). AML patients were diagnosed and classified according to the 2016 World Health Organization (WHO) criteria [19]. Treatment regimens for AML patients were as reported [20, 21]. The detection of gene mutations in this study was described as our previous reports [20, 21]. After informed consents were obtained from all participants, bone marrow (BM) was collected at diagnosed time, and were separated to obtain BM mononuclear cells (BMMNCs) by density-gradient centrifugation using Lymphocyte Separation Medium (Solarbio, Beijing, China) [20, 21]. The current study was approved by the Ethics Committee of Affiliated People’s Hospital of Jiangsu University.

### Targeted bisulfite sequencing

The target gene methylation was detected by Targeted bisulfite sequencing—MethylTarget, which was performed in Genesky Biotechnologies Inc. (Shanghai, China) as our previous investigations [22, 23]. The primers used for *LEP* were shown in Additional file 1: Table S1.

### Reverse transcription and qPCR

Reverse transcription was carried out as our previous studies [20, 21]. The detection of *LEP* mRNA expression was performed by real-time quantitative PCR (qPCR) using AceQ qPCR SYBR Green Master Mix (Vazyme, Piscataway, NJ). The reference gene *ABL1* mRNA, examined by 2 × SYBR Green PCR Mix (Multisciences, Hangzhou, China), was detected to calculate the abundance of *LEP* mRNA expression. The qPCR primers used for *LEP* expression detection were listed in Additional file 1: Table S1. Relative *LEP* mRNA expression was calculated using 2^−∆∆CT^ method.

### Bisulfite modification and qMSP

Genomic DNA was bisulfite converted as our previous reports [21, 22]. The detection of *LEP* methylation level was evaluated by qMSP with primers shown in Additional file 1: Table S1. The reference gene *ALU* methylation level was also detected. Relative *LEP* methylation level was calculated using 2^−∆∆CT^ method.

### Bioinformatics analysis and bioinformatics prediction of miRNA targets

Differential expression analysis for RNA/miRNA sequencing data was calculated using the raw read counts with the R/Bioconductor package “edgeR”, all analyses were controlled for the false discovery rate (FDR) by the Benjamini–Hochberg procedure [24]. The miRNA which could target *LEP* was identified by the venn analysis (http://bioinformatics.psb.ugent.be/webtools/Venn/) of three websites miRDB (http://mirdb.org/miRDB/), miRWalk (http://mirwalk.umm.uni-heidelberg.de/) and TargetScan (http://www.targetscan.org/vert_72/) [25, 26]. All basic statistical analyses were performed using the base functions in R version 3.4 (https://www.r-project.org).

### Statistical analyses

SPSS 20.0 and GraphPad Prism 5.0 were conducted to perform statistical analyses. Mann–Whitney’s U/Kruskal–Wallis followed by Dunn’s post hoc test and Pearson’s *χ*^2^/Fisher’s exact test were used for the comparison of continuous and categorical variables, respectively. Correlation analysis between *LEP* methylation and methylation/expression was performed by Spearman test. The Receiver operating characteristic (ROC) curve and area under the ROC curve (AUC) were used to evaluate *LEP* methylation level in distinguishing AML from controls. Complete remission (CR) was evaluated after 1–2 course of chemotherapy. Overall survival (OS) and leukemia free survival (LFS) were defined as previous report [20]. Survival analysis regarding the effect of *LEP* methylation on OS and LFS was analyzed by Kaplan–Meier analysis and Cox regression analysis (univariate and multivariate). A two-sided *P* less than 0.05 was seen as statistically significant.

## Results

### Identification of prognostically obesity-related genes correlated with DNA methylation in AML

**Fig. 1:**
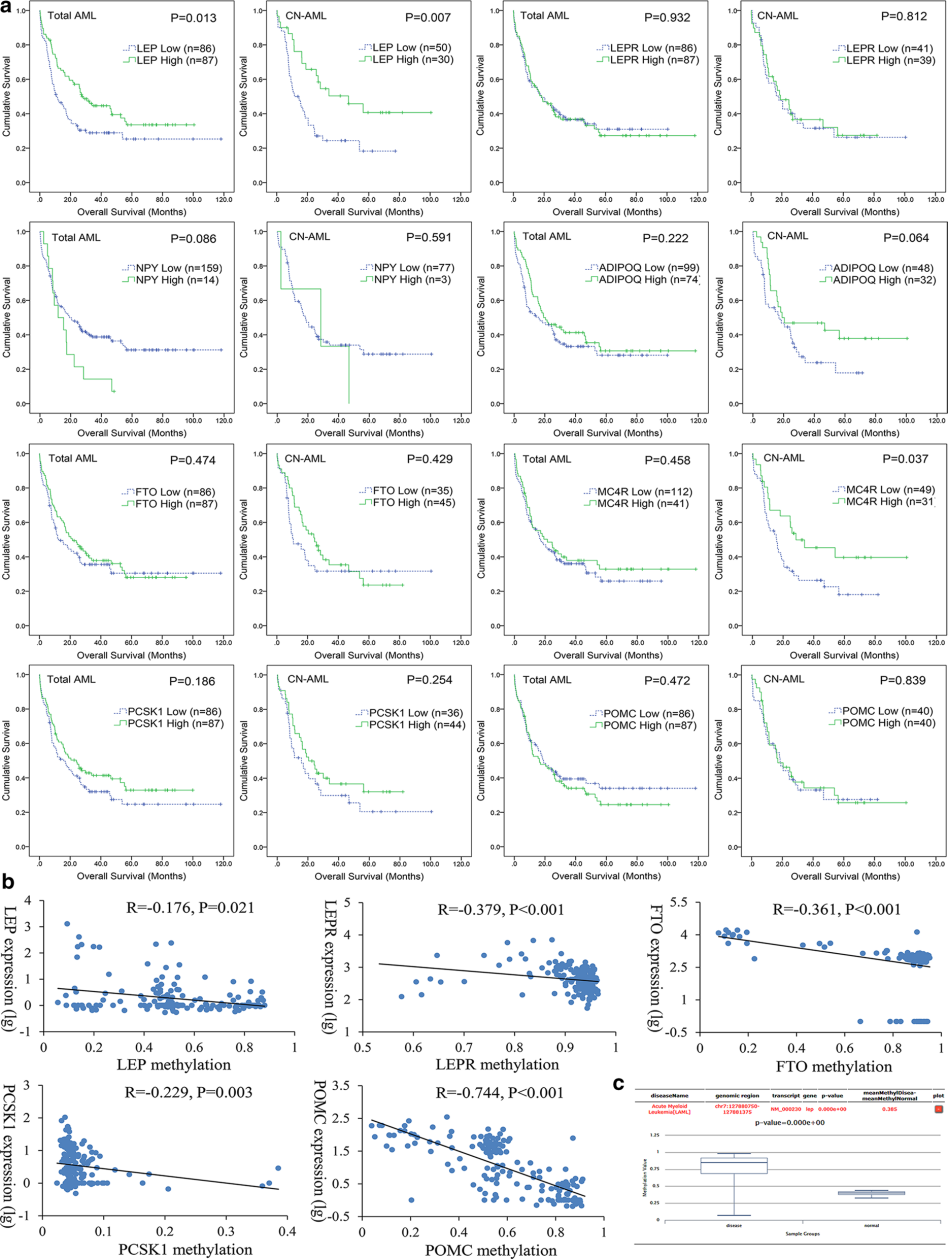
Identification of prognostically obesity-related genes correlated with DNA methylation in AML. **a** The impact of obesity-related genes expression on overall survival among AML patients from TCGA databases. AML patients were divided into two groups by the median methylation level of each gene respectively. **b** Correlation between obesity-related genes expression and methylation among AML patients from TCGA databases. The correlation analysis was conducted by Spearman test. **c** *LEP* methylation level in AML patients and controls obtained by bioinformatics analysis. *LEP* promoter CpG island methylation level was obtained through the human disease methylation database DiseaseMeth version 2.0 (http://bio-bigdata.hrbmu.edu.cn/diseasemeth/analyze.html). *TCGA* The Cancer Genome Atlas

We first used TCGA data to identify the prognostically obesity-related genes including *LEP*, *LEPR*, *NPY*, *ADIPOQ*, *FTO*, *MC4R*, *PCSK1*, and *POMC* in AML. Prognostic value of these genes was analyzed in two groups divided by the median expression level of each gene respectively. In total AML and cytogenetically normal AML (CN-AML) patients, Kaplan–Meier analysis showed that only *LEP* expression was positively associated with OS (*P* = 0.013 and 0.007, Fig. 1a) and LFS (*P* = 0.025 and 0.062, Additional file 2: Figure S1), suggesting the prognostic effect of *LEP* expression in AML.

DNA methylation plays a crucial role in regulating gene expression. We next investigated the association between these obesity-related gene expression and methylation in AML. Among the eight genes, methylation data was available for *LEP*, *LEPR*, *FTO*, *PCSK1*, and *POMC*. Significantly negative association was shown in *LEP* (*R* = − 0.176, *P* = 0.021), *LEPR* (*R* = -0.379, *P* < 0.001), *FTO* (*R* = − 0.361, *P* < 0.001), *PCSK1* (*R* = − 0.229, *P* = 0.003), and *POMC* (*R* = − 0.744, *P* < 0.001) genes (Fig. 1b). These data suggested *LEP*, *LEPR*, *FTO*, *PCSK1*, and *POMC* genes methylation may play main roles in regulating gene expression during leukemogenesis, while *LEP* showed a very weak association. Moreover, we further identified that *LEP* promoter CpG island was hypermethylated in AML by using the DiseaseMeth version 2.0 (*P* < 0.001, Fig. 1c).

### Abnormal LEP promoter methylation in AML by targeted bisulfite sequencing

**Fig. 2:**
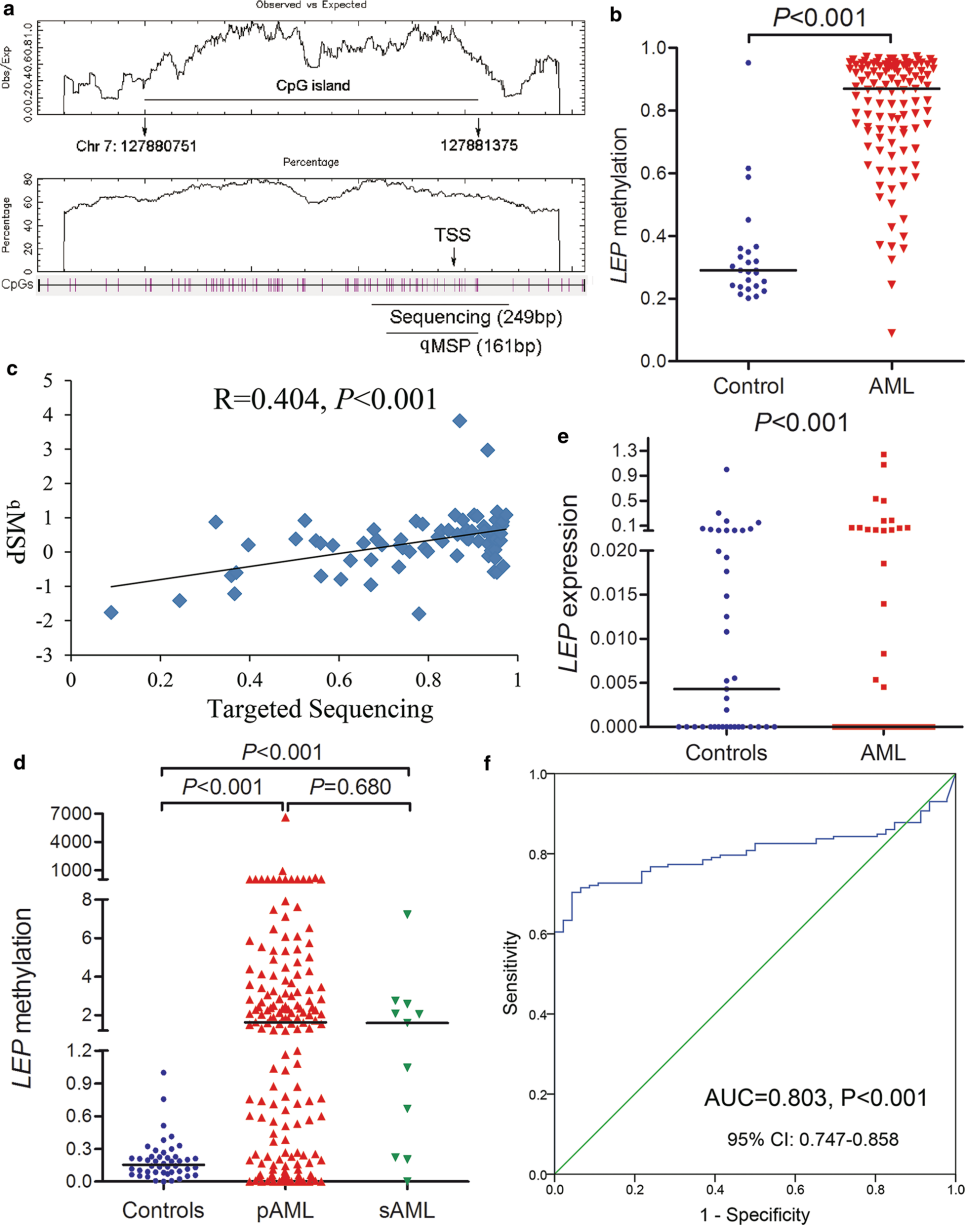
Validation and confirmation of *LEP* methylation in AML. **a** The genomic coordinates (GC) of *LEP* promoter region CpG island and primer locations. The panel plots the GC content as a percentage of the total. Each vertical bar in the bottom panel represents the presence of a CpG dinucleotide. Black horizontal lines indicate regions amplified by sequencing primer pairs and qMSP primer pairs. CpGplot (http://emboss.bioinformatics.nl/cgi-bin/emboss/cpgplot) and Methyl Primer Express v1.0 software were used for creating the figure. *TSS* transcription start site, *qMSP* real-time quantitative methylation-specific PCR; **b** *LEP* methylation level in controls and AML patients detected by targeted bisulfite sequencing. **c** Correlation between targeted bisulfite sequencing and qMSP results for *LEP* methylation in AML patients. The correlation analysis was conducted by Spearman test. **d** *LEP* methylation level in controls and AML patients examined by qMSP. AML included de novo AML and sAML which indicated MDS-derived AML. **e** *LEP* expression level in controls and AML patients. *LEP* expression level was examined by qPCR. **f** ROC curve analysis of *LEP* methylation distinguishing AML from controls

To validate the methylation pattern of *LEP* in AML, we analyzed CpG island methylation located at the *LEP* promoter region (Fig. 2a) by targeted bisulfite sequencing in BMMNCs samples of 25 controls and 111 de novo AML patients. The sequencing mean bait coverage attached 1694 ×, and Q30 was 75.56% [22, 23]. The targeted sequencing results exhibited that the level of *LEP* methylation in AML patients was markedly higher than that in controls (*P* < 0.001, Fig. 2b).

### Further confirmation of LEP methylation in a larger cohort of AML by qMSP

In order to explore whether *LEP* methylation could be helpful utilized in patients diagnosis, prognosis and risk/treatment assessment, we further expanded the patients samples including 46 controls, 161 primary AML and 11 secondary AML to explore clinical implication of *LEP* methylation by using a more rapid and convenient methodology—qMSP. The primers for qMSP were designed located inside the sequencing primer (Fig. 2c), and the results analyzed by qMSP results was positively associated with the results by targeted bisulfite sequencing (*R* = 0.404, *P* < 0.001, Fig. 2c). In addition, *LEP* promoter hypermethylation in primary and secondary AML was further confirmed by qMSP (both *P* < 0.001, Fig. 2d). However, *LEP* methylation showed no significant difference between primary and secondary AML (*P* = 0.680, Fig. 2d). We next detected *LEP* expression in controls and AML patients with available RNA samples by qPCR. *LEP* expression was significantly decreased in AML (*P* < 0.001, Fig. 2e), and was inversely correlated with *LEP* methylation (*R* = − 0.338, *P* = 0.009, *n* = 59, Spearman test).

### Clinical implication of LEP methylation in AML

ROC curve analysis exhibited that *LEP* promoter methylation may be severed as an underlying biological marker for distinguishing AML from controls with an AUC of 0.803 (95% CI 0.747–0.858, *P* < 0.001, Fig. 2f). By the ROC analysis, *LEP* methylation at the value of 1.011 was set as cutoff point due to the sensitivity was 60.5% and the specificity was 100%. According to the set point, we divided AML patients into two groups to analyze the clinical significance of *LEP* methylation. No significant differences were found between two groups with regard to age, white blood cells, and hemoglobin (*P* > 0.05, Table 1). However, *LEP* hypermethylation tended to be associated with male patients and higher BM blasts (*P* = 0.057 and 0.064, respectively, Table 1), and significantly correlated with lower platelets (*P* = 0.046, Table 1). Moreover, there were significant differences between two groups in the distribution of French-American-British (FAB) classifications and karyotypes (*P* = 0.044 and 0.042, respectively, Table 1). *LEP* hypermethylation was less frequently occurred in M3/t(15;17) subtypes (*P* = 0.003 and 0.001, respectively). Moreover, there were no significant associations between *LEP* hypermethylation and gene mutations besides *N/R-RAS* mutations with a trend (*P* = 0.098, Table 1).

**Table 1:** Comparison of clinical and laboratory features between *LEP* hypermethylated and non-hypermethylated AML patients

Patient’s features	Non-hypermethylated (*n* = 68)	Hypermethylated (*n* = 104)	*P* value
Sex, male/female	35/33	69/35	0.057
Median age, years (range)	55.5 (18–85)	55 (18–86)	0.872
Median WBC, × 10^9^/L (range)	12.5 (0.9–528.0)	18.4 (0.3–232.1)	0.626
Median hemoglobin, g/L (range)	76 (32–147)	79 (32–144)	0.951
Median platelets, × 10^9^/L (range)	50 (6–447)	38.3 (3–415)	0.046
Median BM blasts, % (range)	45 (5.5–97.5)	56.5 (1–99)	0.064
FAB classifications			0.044
M0	0	2	
M1	5	6	
M2	21	45	
M3	19	10	
M4	15	20	
M5	7	14	
M6	1	5	
No data	0	2	
Karyotypes			0.042
Normal	24	52	
t(8;21)	4	8	
inv(16)	0	2	
t(15;17)	19	8	
+8	2	3	
-5/5q-	1	0	
-7/7q-	0	1	
t(9;22)	1	1	
11q23	0	2	
Complex	7	10	
Others	7	9	
No data	3	8	
Gene mutations			
* CEBPA* (±)	4/53	12/69	0.187
*NPM1* (±)	5/52	10/71	0.587
*FLT3*-ITD (±)	4/53	8/73	0.761
*C-KIT* (±)	5/52	3/78	0.274
*N/K-RAS* (±)	3/54	12/69	0.098
*IDH1/2* (±)	3/54	2/79	0.404
*DNMT3A* (±)	2/55	6/75	0.470
*U2AF1* (±)	0/57	3/78	0.267
*SRSF2* (±)	3/54	1/80	0.306
*SETBP1* (±)	0/57	2/79	0.512
CR, total AML (±)	33/26	29/56	0.011
CR, non-M3 AML (±)	20/22	23/56	0.049
CR, CN-AML (±)	11/9	14/29	0.105

Patients’ blasts less than 20% with t(15;17) cytogenetic aberrations

*WBC* white blood cells, *BM* bone marrow, *FAB* French-American-British classification, *CR* complete remission

### LEP methylation was associated with prognosis in AML

Firstly, we revealed the significant association of *LEP* methylation with CR rate in AML patients. Notably, CR rate in *LEP* hypermethylated patients was significantly lower than that in *LEP* non-hypermethylated patients among whole-cohort AML and non-M3 AML (*P* = 0.011 and 0.049, respectively, Table 1). In CN-AML, we did not observe the significant difference for CR between *LEP* hypermethylated and non-hypermethylated patients (*P* = 0.105, Table 1). Since the significant associations of *LEP* methylation with CR were observed among whole-cohort AML and non-M3 AML, Logistic regression analysis was performed to confirm the effect of *LEP* methylation on CR. After adjusting for the well-known prognostic factors, *LEP* hypermethylation acted as an independent risk factor negatively affecting CR in both whole-cohort AML and non-M3 AML patients (*P* = 0.017 and 0.015, respectively, Tables 2 and 3).

**Table 2:** Logistic regression analyses of variables for complete remission in AML patients

Variables	Univariate analysis		Multivariate analysis	
	Odds ratio (95% CI)	*P* value	Odds ratio (95% CI)	*P* value
*LEP* methylation	0.408 (0.206–0.807)	0.010	0.371 (0.164–0.837)	0.017
Age	0.151 (0.067–0.337)	0.000	0.142 (0.058–0.343)	0.000
WBC	0.284 (0.136–0.593)	0.001	0.545 (0.232–1.280)	0.164
Cytogenetic risks	0.317 (0.178–0.564)	0.000	0.349 (0.190–0.642)	0.001
*NPM1* mutations	1.530 (0.481–4.867)	0.472		
*FLT3*-ITD mutations	0.819 (0.219–3.071)	0.768		
*C-KIT* mutations	2.625 (0.461–14.932)	0.277		
*N/K-RAS* mutations	0.381 (0.098–1.487)	0.165		
*DNMT3A* mutations	0.480 (0.089–2.581)	0.392		
*U2AF1* mutations	Undermined	0.999		
*IDH1/2* mutations	0.827 (0.133–5.141)	0.838		
*SRSF2* mutations	Undermined	0.999		
*SETBP1* mutations	1.255 (0.077–20.556)	0.874		

Variables including *LEP* methylation (hypermethylation vs. non-hypermethylation), age (≤ 60 vs. > 60 years), WBC (≥ 30 × 10^9^ vs. < 30 × 10^9^ /L), and gene mutations (mutant vs. wild-type). Multivariate analysis includes variables with *P* < 0.100 in univariate analysis

**Table 3:** Logistic regression analyses of variables for complete remission in non-M3 AML patients

Variables	Univariate analysis		Multivariate analysis	
	Odds ratio (95% CI)	*P* value	Odds ratio (95% CI)	*P* value
*LEP* methylation	0.452 (0.208–0.982)	0.045	0.330 (0.135–0.806)	0.015
Age	0.215 (0.091–0.509)	0.000	0.191 (0.076–0.479)	0.000
WBC	0.349 (0.157–0.778)	0.010	0.522 (0.215–1.266)	0.151
Cytogenetic risks	0.463 (0.239–0.899)	0.023	0.398 (0.189–0.838)	0.015
*NPM1* mutations	2.005 (0.620–6.488)	0.246		
*FLT3*-ITD mutations	0.764 (0.180–3.251)	0.715		
*C-KIT* mutations	2.458 (0.392–15.426)	0.337		
*N/K-RAS* mutations	0.481 (0.122–1.902)	0.297		
*DNMT3A* mutations	0.605 (0.112–3.286)	0.561		
*U2AF1* mutations	Undermined	0.999		
*IDH1/2* mutations	1.045 (0.167–6.555)	0.962		
*SRSF2* mutations	Undermined	0.999		
*SETBP1* mutations	1.579 (0.096–26.002)	0.749		

Variables including *LEP* methylation (hypermethylation vs. non-hypermethylation), age (≤ 60 vs. > 60 years), WBC (≥ 30 × 10^9^ vs. < 30 × 10^9^ /L), and gene mutations (mutant vs. wild-type). Multivariate analysis includes variables with *P* < 0.100 in univariate analysis

**Fig. 3:**
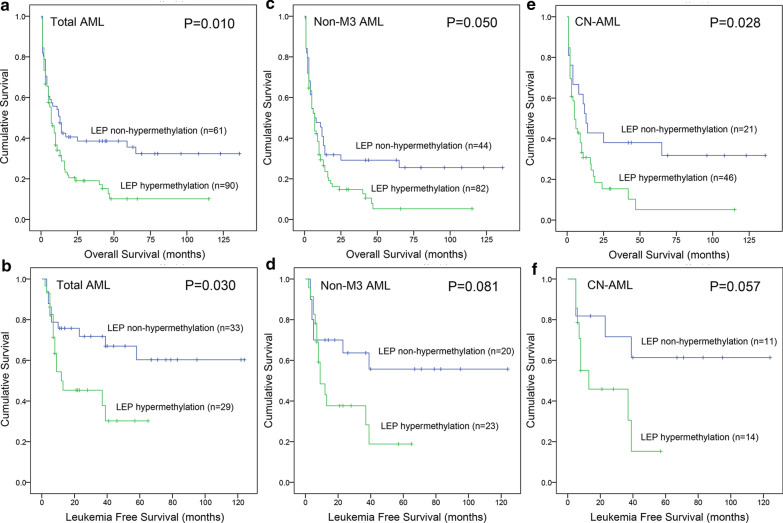
Prognostic value of *LEP* methylation in AML patients. **a**, **c**, **e** The impact of *LEP* methylation on overall survival among whole-cohort AML, non-M3-AML, and CN-AML patients, respectively. **b**, **d**, **f** The impact of *LEP* methylation on leukemia-free survival among whole-cohort AML, non-M3-AML, and CN-AML patients, respectively

Secondly, we also analyzed the effect of *LEP* methylation on OS and LFS in AML patients. Kaplan–Meier analysis indicated that *LEP* hypermethylated patients exhibited shorter OS time than *LEP* non-hypermethylated patients among total AML, non-M3 AML and CN-AML patients (*P* = 0.010, 0.050, and 0.028, respectively, Fig. 3a, c, e). For LFS, significant difference was only observed in total AML between two groups (*P* = 0.030, 0.081, and 0.057, respectively, Fig. 3b, d, f). Furthermore, by Cox regression analysis, *LEP* hypermethylation could severe as a prognostic biomarker independently affecting OS among total AML with a trend (*P* = 0.052, Table 4) and non-M3 AML patients (*P* = 0.041, Table 5).

**Table 4:** Cox regression analyses of variables for overall survival in AML patients

Variables	Univariate analysis		Multivariate analysis	
	Hazard ratio (95% CI)	*P* value	Hazard ratio (95% CI)	*P* value
*LEP* methylation	1.639 (1.104–2.435)	0.014	1.515 (0.996–2.304)	0.052
Age	2.690 (1.839–3.933)	0.000	2.033 (1.364–3.031)	0.000
WBC	2.358 (1.613–3.447)	0.000	1.980 (1.350–2.903)	0.000
Cytogenetic risks	1.723 (1.386–2.143)	0.000	1.427 (1.112–1.831)	0.005
*NPM1* mutations	0.769 (0.371–1.594)	0.479		
*FLT3*-ITD mutations	0.858 (0.396–1.860)	0.699		
*C-KIT* mutations	0.870 (0.319–2.375)	0.785		
*N/K-RAS* mutations	1.097 (0.549–2.192)	0.793		
*DNMT3A* mutations	1.615 (0.745–3.500)	0.225		
*U2AF1* mutations	2.482 (0.771–7.995)	0.128		
*IDH1/2* mutations	0.844 (0.265–2.684)	0.774		
*SRSF2* mutations	2.113 (0.767–5.820)	0.148		
*SETBP1* mutations	0.657 (0.091–4.729)	0.677		

Variables including *LEP* methylation (hypermethylation vs. non-hypermethylation), age (≤ 60 vs. > 60 years), WBC (≥ 30 × 10^9^ vs. < 30 × 10^9^ /L), and gene mutations (mutant vs. wild-type). Multivariate analysis includes variables with *P* < 0.100 in univariate analysis

**Table 5:** Cox regression analyses of variables for overall survival in non-M3 AML patients

Variables	Univariate analysis		Multivariate analysis	
	Hazard ratio (95% CI)	*P* value	Hazard ratio (95% CI)	*P* value
*LEP* methylation	1.496 (0.979–2.287)	0.063	1.584 (1.018–2.464)	0.041
Age	2.015 (1.362–2.981)	0.000	1.802 (1.203–2.700)	0.004
WBC	1.933 (1.302–2.870)	0.001	1.796 (1.209–2.668)	0.004
Cytogenetic risks	1.525 (1.173–1.982)	0.002	1.388 (1.050–1.834)	0.021
*NPM1* mutations	0.646 (0.310–1.346)	0.243		
*FLT3*-ITD mutations	0.905 (0.416–1.967)	0.801		
*C-KIT* mutations	0.757 (0.239–2.402)	0.636		
*N/K-RAS* mutations	0.944 (0.470–1.895)	0.871		
*DNMT3A* mutations	1.420 (0.653–3.088)	0.377		
*U2AF1* mutations	2.293 (0.709–7.413)	0.166		
*IDH1/2* mutations	0.722 (0.226–2.309)	0.583		
*SRSF2* mutations	1.892 (0.685–5.222)	0.218		
*SETBP1* mutations	0.576 (0.080–4.152)	0.584		

Variables including *LEP* methylation (hypermethylation vs. non-hypermethylation), age (≤ 60 vs. > 60 years), WBC (≥ 30 × 10^9^ vs. < 30 × 10^9^/L), and gene mutations (mutant vs. wild-type). Multivariate analysis includes variables with *P* < 0.100 in univariate analysis

### MiRNA signatures correlated with LEP in AML

**Fig. 4:**
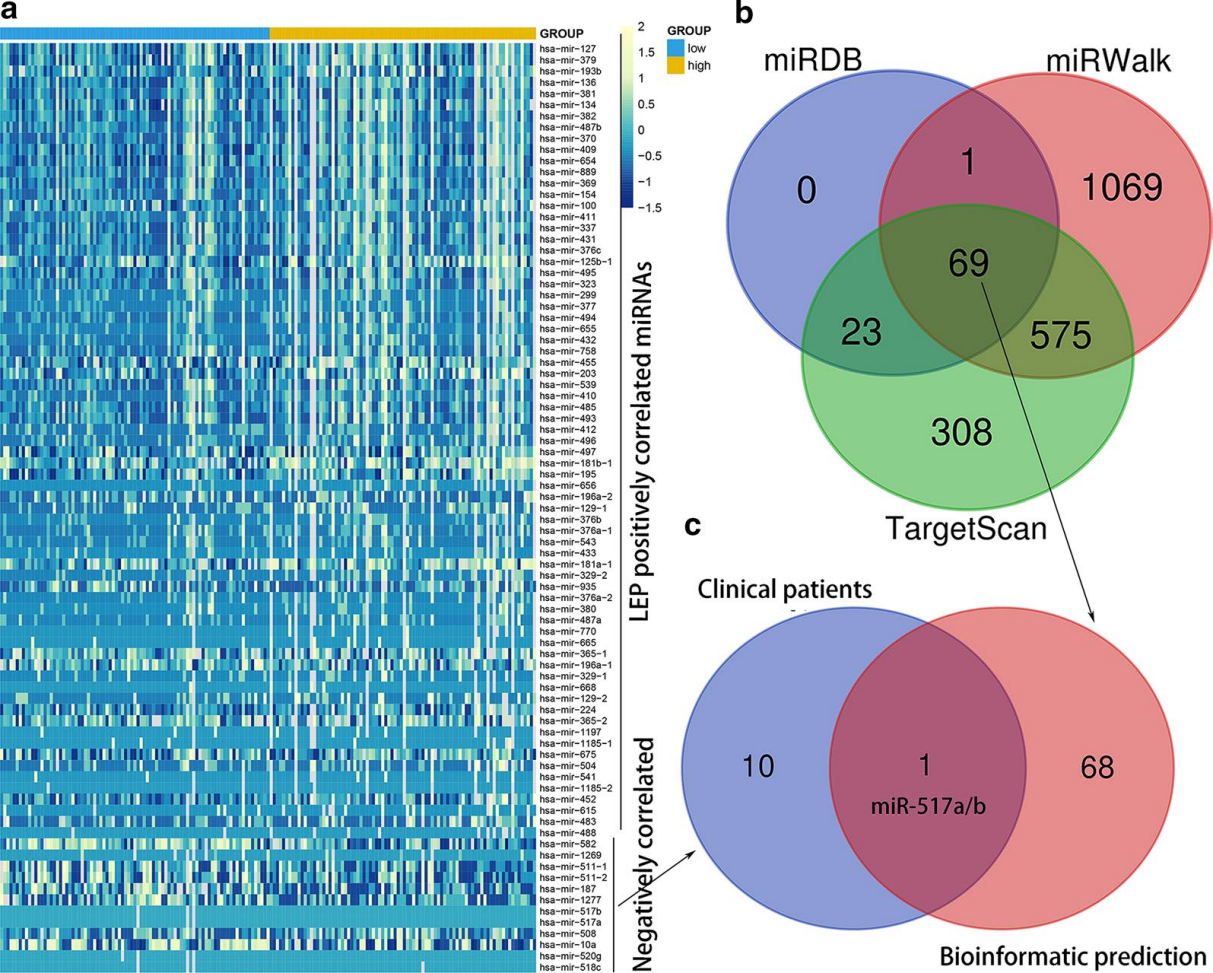
MicroRNA signatures correlated with *LEP* in AML. **a** Expression heatmap of differentially expressed microRNAs between lower- and higher-expressed *LEP* in AML patients among TCGA datasets (FDR < 0.05, *P* < 0.05 and |log2 FC|> 1). **b** Venn results of microRNAs which could target *LEP* predicted by TargetScan (http://www.targetscan.org/vert_72/), miRDB (http://mirdb.org/miRDB/), and miRWalk (http://mirwalk.umm.uni-heidelberg.de/). **c** Venn results of microRNAs shared in the negatively correlated microRNAs in **a** and the bioinformatics prediction in **b**

Due to a very weak correlation of *LEP* expression with *LEP* methylation in AML patients from both TCGA cohort and validation data, we thought that *LEP* expression in AML was not only regulated by *LEP* methylation, and other mechanism also involved such as miRNAs. To gain insights into the molecular signatures associated with *LEP* in AML, we first compared the transcriptomes of miRNAs expression signatures in lower and higher *LEP* expression groups (based on the median level of *LEP* expression) of AML patients from TCGA datasets. A total of 83 differentially expressed miRNAs (included 71 positively correlated and 12 negatively correlated) (FDR < 0.05, *P* < 0.05, |log2 FC|> 1; Fig. 4a; Additional file 3) were identified between two groups. The negatively correlated miRNAs such as *miR-10a* was identified to be significantly associated with AML with *NPM1* mutation [27], whereas the other genes including *miR-582*, *miR-517*, *miR-511*, *miR-508*, m*iR-518c*, *miR-520g*, and *miR-187* were less investigated. Moreover, *LEP* was identified as a direct target of 69 miRNAs by bioinformatics prediction (Fig. 4b, Additional file 4). Of these miRNAs, *miR-517a/b* was shared in both clinical data and bioinformatics prediction, suggesting that *LEP* may be also regulated by *miR-517a/b* expression in AML (Fig. 4c).

## Discussion

Obesity confers enhanced risk for multiple diseases including cancer, and is increasingly recognized as a growing cause of preventable cancer risk [3–6]. The DNA methylation alterations in obesity-related genes have been implicated in several human solid tumors [12, 15]. Previously, promoter methylation of obesity-related genes including *LEP*, *NPY*, and *LEPR* was involved in tumorigenesis of renal cell carcinoma, and *LEPR* methylation was associated with prognosis, and predicted renal cell carcinoma recurrence [12, 28]. Herein, we for the first time evaluated prognostic value of obesity-related gene expression and methylation in AML. By the identification and validation stage, we finally revealed that *LEP* methylation, negatively associated with *LEP* expression, was independently associated clinical outcome in AML.

The expression pattern and direct role of *LEP* remains controversial in AML. Functional studies have showed that leptin presented an oncogenic role in AML biology by affecting cell proliferation and angiogenesis [29–31]. In clinics, although no significant difference of serum leptin concentrations were found between de novo AML patients and controls in two previous reports [32, 33], two independent investigations by Aref et al. and Bruserud et al. showed that serum leptin levels in AML patients were significantly lower than controls and had negative correlation with percentage of BM blasts and white blood cells [34, 35]. In our study, we detected *LEP* mRNA level in BMMNCs but not in serum of AML patients, and were found to be significantly decreased. The decreased expression of *LEP* may be caused by *LEP* promoter methylation in AML cells. In accordance with the previous study, we also observed *LEP* hypermethylation was associated with higher percentage of BM blasts and lower platelets. These results suggested DNA methylation-mediated leptin inactivation was a frequent event in AML cells. The reduction of autocrine of leptin in leukemia cells may negatively feedback regulates the increase of paracrine of leptin from adipose tissues into cancer microenvironment to promote leukemogenesis. Accordingly, further functional studies in vivo and in vitro are required to confirm our hypothesis.

Besides the DNA methylation, miRNAs expression was also identified to be associated with *LEP* expression in AML. In this study, we identified that *LEP* expression may be also regulated by *miR-517a/b* expression. Although few investigations revealed the *miR-517a/b* expression pattern in AML, a number of studies have reported the oncogenic role of *miR-517a/b* in diverse human solid tumors [36–38]. These results suggested that multiple factors were involved in regulating *LEP* expression in AML biology. Obviously, additional studies are required to confirm the direct links of *LEP* with *miR-517a/b* by luciferase assay, and the direct role of *miR-517a/b* in AML needs further functional studies.

## Conclusion

Our findings indicated that the obesity-related gene *LEP* methylation is associated with *LEP* inactivation, and acts as an independent prognostic predictor in AML.

## Supplementary Information

**Additional file 1**. Primers used for MethylTarget sequencing, qPCR and qMSP.

**Additional file 2**. The impact of obesity-related genes expression on leukemia-free survival among AML patients from TCGA databases. 

**Additional file 3**. Different expressed microRNAs between lower and higher *LEP* expression groups.

**Additional file 4**. Venn results of microRNAs targeting *LEP*.

## Data Availability

The datasets used and/or analyzed during the current study are available from the corresponding author on reasonable request.

## Abbreviations

AML: Acute myeloid leukemia
ELN: European LeukemiaNet
BMI: Body mass index
TCGA: The Cancer Genome Atlas
qMSP: Real-time quantitative methylation-specific PCR
WHO: World Health Organization
BM: Bone marrow
BMMNCs: BM mononuclear cells
qPCR: Real-time quantitative PCR
CR: Complete remission
LFS: Leukemia-free survival
OS: Overall survival
CN-AML: Cytogenetically normal AML
FAB: French-American-British
