# Supplementary material for: Identification and validation of obesity-related gene LEP methylation as a prognostic indicator in patients with acute myeloid leukemia
Source: Clin Epigenetics. 2021 Jan 23;13:16. doi: 10.1186/s13148-021-01013-9 (PMC7824952; doi:10.1186/s13148-021-01013-9)
Supplement: Supplementary file 1 — Additional file 1. Primers used for MethylTarget sequencing, qPCR and qMSP. [file 13148_2021_1013_MOESM1_ESM.docx]

**Supplementary material Table S1. Primers used for MethylTarget sequencing, RQ-PCR, and RQ-MSP.**

| Primers | Primer sequence (5’to 3’) |
| --- | --- |
| MethylTarget sequencing |  |
| *LEP-*F | GTGGGGTTTTGTGGTTTGTT |
| *LEP*-R | ACACACTACRAACCCAAAACTAACA |
|  |  |
| RQ-PCR |  |
| *LEP-*F | TTGGCCCTATCTTTTCTATG |
| *LEP*-R | GCATACTGGTGAGGATCTGT |
|  |  |
| RQ-MSP primers |  |
| *LEP*-MF | TCGGGGTTTTATTTTGTAATC |
| *LEP*-MR | CACGTCGCTACCCTAAAA |
| *LEP*-UF | GTTGGGGTTTTATTTTGTAATT |
| *LEP*-UR | ACACATCACTACCCTAAAA |

RQ-PCR: real-time quantitative PCR; RQ-MSP: real-time quantitative methylation-specific PCR
