# Supplementary material for: Identification and validation of obesity-related gene LEP methylation as a prognostic indicator in patients with acute myeloid leukemia
Source: Clin Epigenetics. 2021 Jan 23;13:16. doi: 10.1186/s13148-021-01013-9 (PMC7824952; doi:10.1186/s13148-021-01013-9)
Supplement: Supplementary file 2 — Additional file 2. The impact of obesity-related genes expression on leukemia-free survival among AML patients from TCGA databases. [file 13148_2021_1013_MOESM2_ESM.docx]

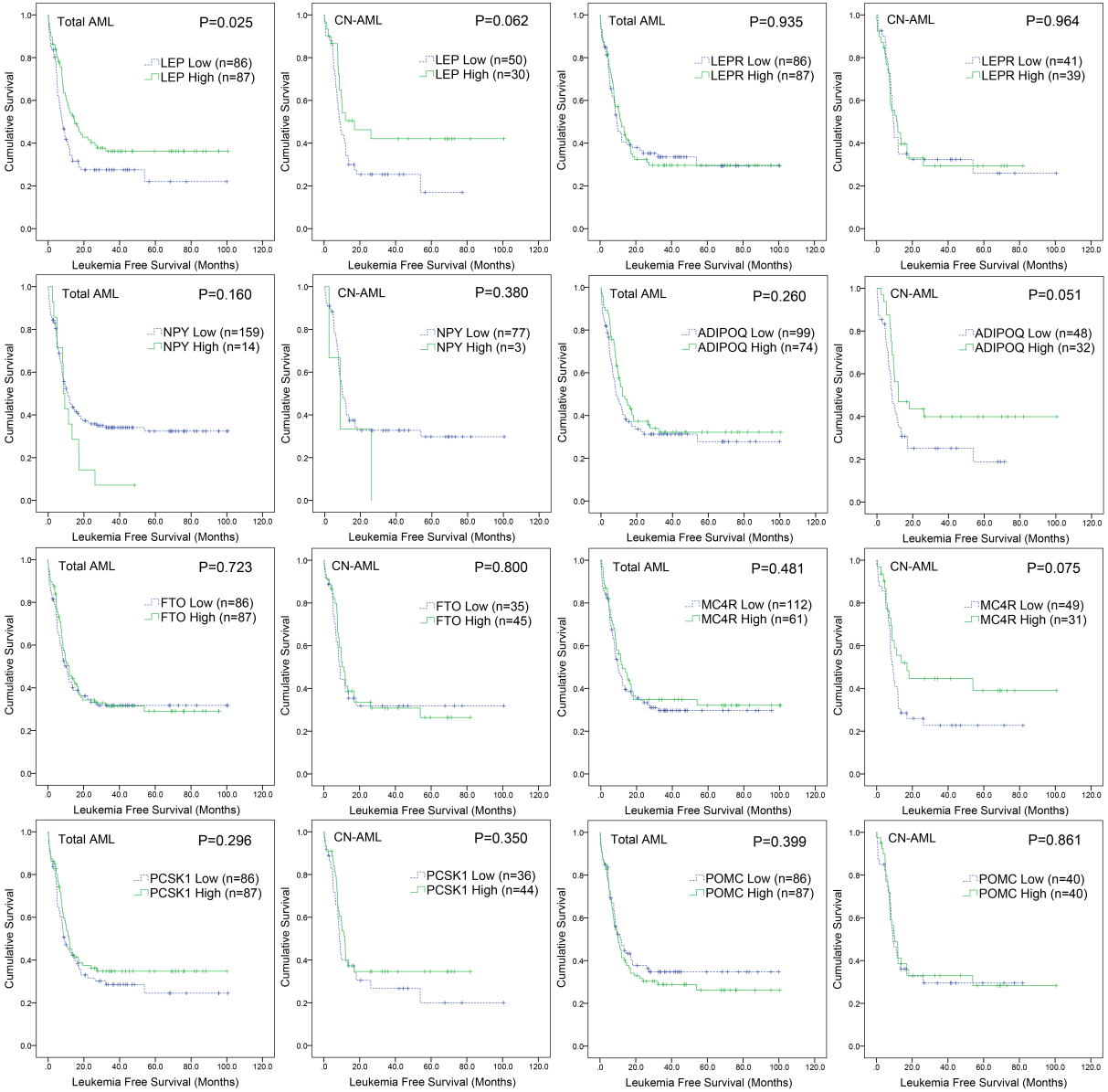


**Figure S1. The impact of obesity-related genes expression on leukemia-free survival among AML patients from TCGA databases.** AML patients were divided into two groups by the median methylation level of each gene respectively. TCGA: The Cancer Genome Atlas; CN-AML: cytogenetically normal AML.
